# Cofactor Metabolic Engineering of *Escherichia coli* for Aerobic L-Malate Production with Lower CO_2_ Emissions

**DOI:** 10.3390/bioengineering10080881

**Published:** 2023-07-25

**Authors:** Zhiming Jiang, Youming Jiang, Hao Wu, Wenming Zhang, Fengxue Xin, Jiangfeng Ma, Min Jiang

**Affiliations:** State Key Laboratory of Materials-Oriented Chemical Engineering, College of Biotechnology and Pharmaceutical Engineering, Nanjing Tech University, Nanjing 211800, China

**Keywords:** L-malate, *Escherichia coli*, NOG system, electron transport chain, cofactors

## Abstract

*Escherichia coli* has been engineered for L-malate production via aerobic cultivation. However, the maximum yield obtained through this mode is inferior to that of anaerobic fermentation due to massive amounts of CO_2_ emissions. Here, we aim to address this issue by reducing CO_2_ emissions of recombinant *E. coli* during aerobic L-malate production. Our findings indicated that NADH oxidation and ATP-synthesis-related genes were down-regulated with 2 g/L of YE during aerobic cultivations of *E. coli* E23, as compared to 5 g/L of YE. Then, E23 was engineered via the knockout of *nuoA* and the introduction of the nonoxidative glycolysis (NOG) pathway, resulting in a reduction of NAD^+^ and ATP supplies. The results demonstrate that E23 (Δ*nuoA*, NOG) exhibited decreased CO_2_ emissions, and it produced 21.3 g/L of L-malate from glucose aerobically with the improved yield of 0.43 g/g. This study suggests that a restricted NAD^+^ and ATP supply can prompt *E. coli* to engage in incomplete oxidization of glucose, leading to the accumulation of metabolites instead of utilizing them in cellular respiration.

## 1. Introduction

L-malate, a four-carbon dicarboxylic acid, is currently used mainly as an acidulant and taste enhancer in the beverage and food industry [1]. Additionally, it has been identified as one of the twelve most promising platform chemicals from biomass by the Department of Energy of the United States [2]. Commercial L-malate is mainly produced via the hydration of either maleate or fumarate using cell bioconversion or enzymatic conversion [3]. Additionally, L-malate can be synthesized from food or non-food renewable feedstocks with microorganisms, such as *Penicillium viticola*, *Aspergillus niger*, *Bacillus coagulans,* and *Escherichia coli* [4,5,6,7,8].

As a well established model bacterium, *E. coli* has been engineered for L-malate production through both aerobic and anaerobic fermentation. Recently, Zhang et al. constructed a recombinant *E. coli* XZ658, which achieved a final L-malate concentration of 34 g/L with a mass yield of 1.06 g of L-malate per g of consumed glucose during the anaerobic phase [9]. Hu et al. rewired the RuBisCO shunt using the ATP-generating carboxylation pathway for L-malate production in *E. coli*. The fixation capability of CO_2_ was effectively enhanced, and the highest reported yield of 1.09 g of L-malate per g of consumed glucose was achieved [10].

Furthermore, some researchers paid much more attention to the aerobic mode for L-malate production via the Krebs cycle and glyoxylate shunt. Moon et al. constructed a recombinant *E. coli* that was deficient in *pta* and overexpressed the *pckA* gene, resulting in accumulation of 9.62 g/L of L-malate with a yield of 0.61 g of L-malate per g of consumed glucose through aerobic cultivation [7]. Gao et al. employed an in vitro modular pathway optimization strategy in conjunction with in vivo multiplexed combinatorial engineering to engineer a recombinant, which yielded 0.63 g of L-malate per g of consumed glucose [11]. Similarly, Trichez et al. achieved a yield of 0.61 g of L-malate per g of consumed glucose by inactivating *mdh*, *mqo*, *maeAB*, *iclR,* and *arcA*, and this resulted in the expression of PEP carboxylase and NADH-insensitive citrate synthase in *E. coli* [12]. Guo et al. adopted a strategy to enhance the production of L-malate by targeting the reductive tricarboxylic acid (rTCA) pathway to the periplasm, and the resultant engineered strain produced 25.9 g/L of L-malate with a yield of 0.39 g of L-malate per g of consumed glucose [13]. In addition, Li et al. constructed an engineered *E. coli* for L-malate production from xylose, which achieved a titer of 5.90 g/L with a yield of 0.80 g of L-malate per g of xylose [14]. Soto-Varela et al. identified the enzymatic bottlenecks for the aerobic production of L-malate through the systematic gene overexpression of anaplerotic enzymes in *E. coli* and achieved 57.5% of the theoretical maximum molar yield [15].

**Figure 1:**
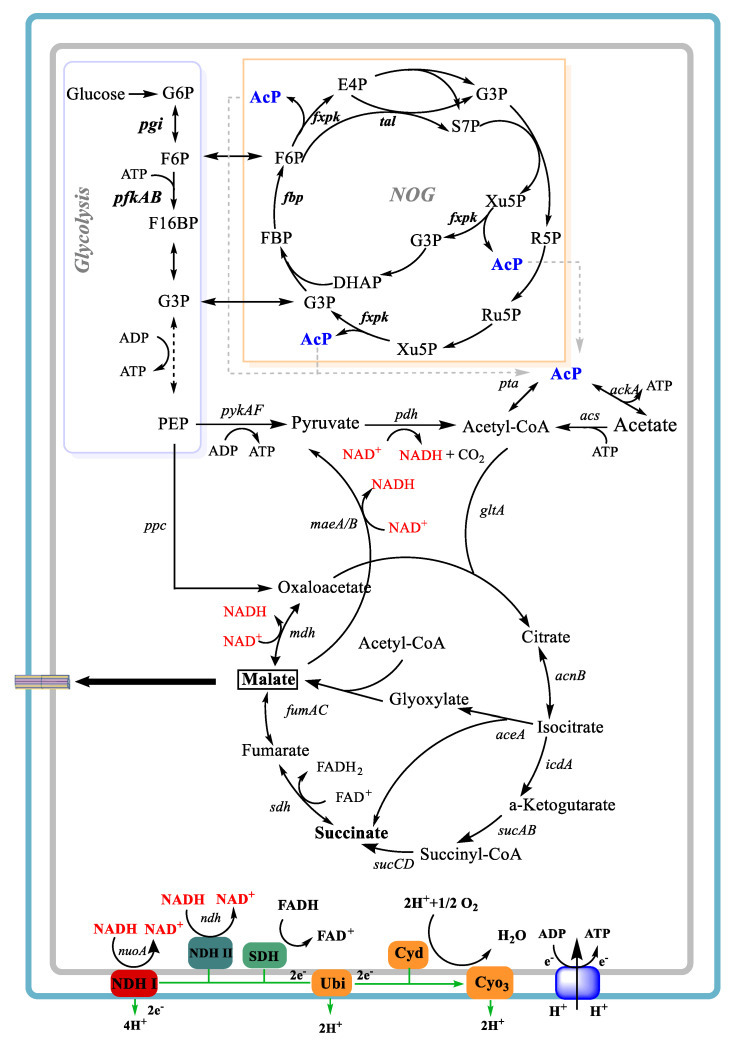
A schematic representation of aerobic L-malate production from glucose by an *E. coli* recombinant.

Despite the construction of several recombinants for the aerobic production of L-malate, the maximum yield achieved was inferior to that attained through anaerobic fermentation. This can be attributed to the substantial CO_2_ emissions, resulting from the dicarboxylic reactions under aerobic conditions (as illustrated in Figure 1). While CO_2_ utilization is one of the hot topics of bioengineering, and many researchers are working on it [16], it is equally crucial to mitigate CO_2_ emissions in certain industrial processes, such as in the fermentation industry. Recently, Bogorad et al. have designed a nonoxidative glycolysis (NOG) pathway, which uses a Xpk-dependent cleavage of sugar phosphates and carbon rearrangement cycles to generate acetyl-CoA without carbon loss [17]. The NOG pathway bypasses the initial C3 formation and directly generates stoichiometric amounts of C2 metabolites without CO_2_ emissions, while requiring one ATP (as shown in Figure 1). In addition, our previous study has established a novel L-malate synthesis routine termed “L-malate overflow metabolism” in *E. coli* AFP111, which utilizes succinate as a carbon source by reducing the efficiency of the electron transport chain (ETC) [18].

In this study, our objective was to expand the L-malate production method by using a recombinant *E. coli* strain with glucose as the aerobic carbon source. Firstly, aerobic cultivations supplemented with varying concentrations of yeast extract (YE) were carried out with *E. coli* E23 to examine the metabolic characteristics and transcriptional level of ETC-related genes. Then, engineered E23 was obtained based on reducing the NAD^+^ and ATP supply through the knockout of *nuoA* and the introduction of the NOG system. Finally, the metabolic characteristics of E23 (Δ*nuoA*, NOG) were evaluated to attain a more accurate comprehension of the provision of NAD^+^ and ATP in the context of aerobic organic acid synthesis.

## 2. Materials and Methods

### 2.1. Strains and Plasmids

The parental strain *E. coli* E23 (BL21 (DE3), *ppc*:trc, *aceBAK*:trc, Δ*maeAB*) was provided by Professor Tao Chen from Tianjin University. This strain was engineered from *E. coli* E2 [19], whose activity of isocitrate dehydrogenase (ICDH) was significantly decreased after long-term adaptive evolution in minimal medium with glycerol as the sole carbon source. In addition, the native promoters of *aceBAK* and *ppc* were replaced with the trc promoter, and the NAD^+^-dependent and NADP^+^-dependent malic enzymes (Encoded by *maeAB*) were both inactivated. *E. coli* DH5α strain was used as the host strain for plasmid construction. All the used and constructed strains are listed in Table 1. The CRISPR-Cas9 system, including the plasmids Pcas9A and pTargetF, was adopted to knockout the chromosome gene, and plasmid pTrc99a was used to overexpress the NOG related genes.

### 2.2. Media and Cultivation Conditions

*E. coli* was grown in Luria-Bertani (LB) broth or agar plate (2%, *w*/*v*) for inoculum preparation and plasmid construction. When necessary, antibiotics were added to the culture medium at the following concentrations: 100 µg/mL of ampicillin (Amp) and 50 μg/mL of spectinomycin (Spec).

The seed culture was prepared by inoculating 20 μL of glycerol cell stock into a 25 mL tube containing 5 mL LB medium and cultivated at 37 °C and 200 rpm. After 12~14 h, 5 mL seed culture was transferred into 100 mL of fresh medium in a 1000 mL baffled shake flask, and it was cultivated for 6~8 h at 37 °C in a rotary shaker at 200 rpm.

Flask cultivations were carried out in a 500 mL flask with 200 mL M9 mineral salt medium at 37 °C and 200 rpm in a rotary shaker supplemented with 20 g/L glucose and YE. Na_2_CO_3_ was used as the CO_2_ source and the buffering agent at 10 g/L. To examine the influence of YE on cell growth and metabolism, YE was varied within the range of 0 to 5 g/L. The cells were cultivated at 37 °C in a rotatory shaker at 200 rpm. When OD_600_ reached 1, 0.05 mM IPTG was added into the medium to induce the expression of genes under control of IPTG-inducible promoter, followed by an additional 48 h incubation at 30 °C. All the flask cultivation experiments were performed in triplicate.

For fed-batch culture, 100 mL inoculum was transferred into a 5-L bioreactor containing 2 L initial cultivation medium composed of M9 minimal medium, 2 g/L YE, and approximately 25 g/L glucose. Temperature and pH were maintained at 37 °C and above 6.8 with 30% (*w*/*v*) Na_2_CO_3_. The aeration rate was maintained around 1 L/min, and dissolved oxygen (DO) was controlled above 10% by controlling the stirrer speed between 200 and 800 rpm. When the glucose concentration was below 5 g/L, concentrated glucose was supplemented into the reactor to approximately 25 g/L. Samples were periodically collected to determine cell growth, as well as glucose and extracellular metabolite concentrations.

### 2.3. Construction of a NDH-1 Defective Mutant

The *nuo* operon, encoding NDH-1, consists of 14 genes, and a NDH-1-defective mutant E23 (Δ*nuoA*) was constructed by deleting the *nuoA* gene, encoding NADH-quinone oxidoreductase subunit A using CRISPR-Cas9 [20,21]. The primers used for *nuoA* knockout and identification are given in Supplementary Table S1. Target-S and Target *nuo*-A were used to construct the target plasmid pTarget T2, and *nuo*-5-S, *nuo*-5-A, *nuo*-3-S, and *nuo*-3-A were used to amplify the donor DNA homologous to the targeting genome loci of *nuoA*. Finally, *nuo*-V-S and *nuo*-V-A were used to certify whether *nuoA* was deleted.

### 2.4. Construction of NOG Expression System

Two key enzymes—bifunctional phosphoketolase (FXPK, encoded by *fxpk* from *Bifidobacterium adolescentis*) and fructose-1,6-bisphosphatase (FBP, encoded by *fbp* from *E. coli*)—were overexpressed to construct the NOG expression system. Firstly, the *fxpk* gene was amplified from *B. adolescentis* genomic DNA and digested by *EcoR* I and *Sal* I, and then it was ligated to the expression vector pTrc99a to generate pTrc-*fxpk*. Then, the *fbp* gene was amplified from *E. coli* genomic DNA and inserted into pTrc-*fxpk* at the *Xba* I restriction site to generate pTrc-*fxpk*-*fbp*, which was transformed to E23 (Δ*nuoA*) to obtain a recombinant E23 (Δ*nuoA*, NOG). The primers used for the construction of pTrc-*fxpk*-*fbp* are given in Supplementary Table S2. SDS-PAGE protein electrophoresis of recombinants of *E. coli* E23 is given in Supplementary Figure S1.

### 2.5. Cell Biomass and Residual Glucose Concentration

Cell growth was monitored by measuring the optical density (OD) at 600 nm with a spectrophotometer (1 OD_600_ = 0.40 g/L DCW). The glucose concentration in the broth was detected by a glucose bioanalyzer (SenSep, Nanjing Membrane Material Industry Technology Research Institute Co., Ltd., Nanjing, China), and Prussian blue (PB) enzyme biosensor chips with nanocubic crystals were manufactured by screen printing technology for the detection of glucose [22,23].

### 2.6. Metabolites Analysis and Yield Analysis

Organic products were determined by high performance liquid chromatography (HPLC) (Chromeleon server monitor, P680 pump, Dionex, Sunnyvale, CA, USA) with a UVD 170U ultraviolet detector at a wavelength of 215 nm and ion exchange chromatographic column (Aminex HPX-87H, 300 mm × 7.8 mm; Hercules, CA, USA). The flow rate was controlled at 0.5 mL/min with the mobile phase of 2.55 mM H_2_SO_4_. L-malate mass yield (g/g) was calculated as the gram accumulated L-malate per gram of consumed glucose.

### 2.7. Determination of CO_2_ Emissions

CO_2_ emissions were determined using an on-line COZIR Wide Range CO_2_ analyzer (GSS, Cumbernauld, UK), whose detection range was 0 to 100%. To reduce the interference caused by vapour, the off-gas was connected with a 500 mL bottle, which was filled with silica gel drier to absorb the vapour. pCO_2_ is defined as the CO_2_ concentration in the exhaust gases (%).

### 2.8. Phosphoketolase Activity Assay

Samples were prepared by shake flask cultivation, and 10 mL samples were centrifuged at 4100 rpm and 4 °C for 10 min at 24 h. Then, they were washed twice with 5 mL of 50 mM cold Tris-HCl buffer (pH 7.5), and enzymatic activity was assayed by measuring the amount of ferric acetyl hydroxamate produced, using the method described previously [24]. 

### 2.9. Quantification of Intracellular NAD(H)

Samples were harvested by centrifugation at 14,000 rpm and 4 °C for 5 min. After carefully removed the supernatant, 300 µL 0.2 mol/L NaOH or 300 µL 0.2 mol/L HCl were separately added to resuspend the pellets for NADH or NAD^+^ analysis. The intracellular concentrations of NAD^+^ and NADH were assayed according to the cycling method described previously [25].

### 2.10. Quantification of Intracellular Acetyl-coA and ATP

10 mL samples were taken into precooled centrifuge tubes and centrifuged at 12,000 rpm and 4 °C for 10 min and subsequently washed twice with 30 mL 100 mM sodium phosphate buffer (pH 3.0) at 4 °C, after which the cell pellets were stored at −80 °C. Acetyl-coA was measured according to the method of Yang et al. [26].

1 mL of cold 30% (*w*/*v*) trichloroacetic acid was added to the samples (4 mL) and mixed thoroughly. The ATP concentrations were then measured using the BacTiter-Glo^TM^ Microbial Cell Viability Assay Kit (Promega, Madison, Wisconsin, USA).

### 2.11. Transcriptional Analysis

Transcriptional levels of the related genes were analyzed by RT-qPCR using an ABI 7500 Real-Time PCR system with SYBRs PemixExTaqTM kit (Takara, Shiga, Japan). During the comparative transcriptional analysis of ETC of E23 with 2 g/L and 5 g/L YE, we collected the samples at the mid-logarithmic growth phase. The samples were centrifuged at 14,000 rpm for 5 min at 4 °C, and the total RNA was extracted from the precipitate by a commercial kit (Takara, Japan). Then, the cDNA synthesis kit (Takara, Japan) was used to reverse transcribe the RNA under the manufacturer’s recommendation. The RT-qPCR reactions used the ABI 7500 Real-Time PCR system. The 16 s rRNA gene was used as an internal control for the RT-qPCR determinations. 

## 3. Results

### 3.1. Metabolic Characteristics of E. coli E23 with Different Concentrations of YE

**Figure 2:**
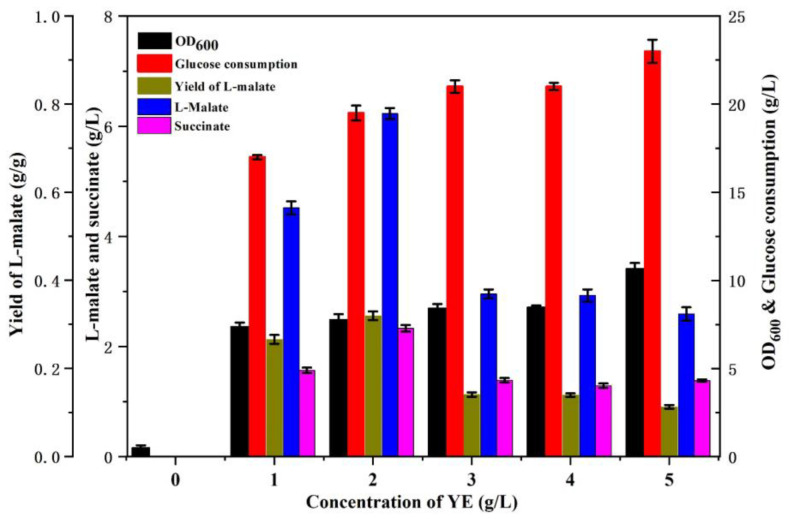
The growth and metabolic characteristics of *E. coli* E23, supplemented with different concentrations of YE.

Previously, *E. coli* E23, deficient in malic enzyme genes *maeA*, *maeB,* and constitutive expression of phosphoenol pyruvate carboxylase gene *ppc* and glyoxylate pathway gene *aceBAK*, was considered as a potential candidate for aerobic L-malate production. Firstly, the metabolic characteristics of *E. coli* E23 were investigated under different nitrogen concentrations. As shown in Figure 2, E23 suffered growth restriction with M9 medium without organic nitrogen. The addition of YE was found to have a restorative effect on cell growth and glucose consumption, in tandem with an increasing concentration of YE. However, the production of organic acids did not exhibit a linear relationship with the YE concentration. Specifically, the highest accumulation of L-malate or succinate was observed when 2 g/L YE was supplemented, as compared to lower or higher nitrogen concentration. It indicated that cultivation with a relatively lower concentration of YE was beneficial for organic acid accumulation during aerobic cultivation.

### 3.2. Transcriptional Analysis of E. coli E23 with Different Concentrations of YE

**Figure 3:**
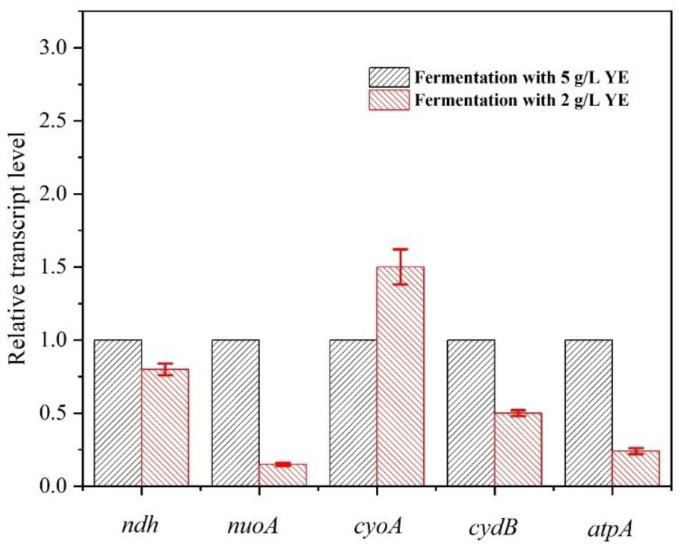
Comparative transcriptional analysis of ETC components of *E. coli* E23 in response to fermentations with 5 g/L and 2 g/L YE.

In a previous study, we observed that nitrogen limitation was one critical factor provoking the L-malate overflow metabolism by down-regulating the ETC efficiency of *E. coli* AFP111 with succinate as carbon source [18]. Here, a comparative transcriptional analysis of the ETC in E23 was carried out. As shown in Figure 3, the transcription of *ndh*, *nuoA*, *cydB,* and *atpA* genes with 2 g/L YE were down-regulated by 0.20-, 0.85-, 0.50-, and 0.76-fold, respectively, compared with those with 5 g/L YE. The transcriptional down-regulation of these genes would significantly reduce the ETC efficiency, especially the NADH oxidation and the ATP synthesis, as *nuoA* and *atpA* were the prominent down-regulated genes. In addition, the transcriptional up-regulation of *cyoA* by 52% has been observed to facilitate the pumping of protons across the membrane, leading to a decrease in intracellular ATP production. Additionally, the decreased intracellular H^+^ can inhibit the reduction of NAD^+^ to NADH, and thus it can down-regulate the reaction from malate to oxaloacetate.

### 3.3. Engineering E. coli E23 to Decrease NAD^+^ Supply by the Knockout of nuoA

**Figure 4:**
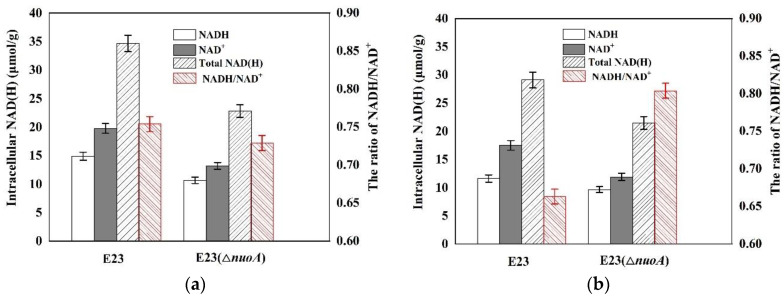
Intracellular concentration and ratio of NAD(H) at the beginning (**a**) and end of the stationary phase (**b**) of growth in *E. coli* E23 and *E. coli* E23 (Δ*nuoA*).

In the respiratory system, the first reactions of NADH oxidation for the electron transport chain are catalyzed by membrane-bound NADH dehydrogenase I (encoded by *nuo* operon) and NADH dehydrogenase II (encoded by *ndh*), which have a significant impact on the ETC efficiency [27]. Here, the gene *nuoA* was deleted in E23 by the Crispr-cas9 method to investigate whether the cells would be under a NAD^+^-limited environment. Thus, the concentrations of intracellular NADH and NAD^+^ at the beginning (Figure 4a) and the end (Figure 4b) of the stationary growth phase of E23 and E23 (Δ*nuoA*) were measured. As a result, the knockout of *nuoA* resulted in a dramatic drop in the NAD^+^ and NADH concentration, and, thus, the NAD(H/+) pool was rather low. In addition, the NADH/ NAD^+^ ratios were always lower than 1.

**Figure 5:**
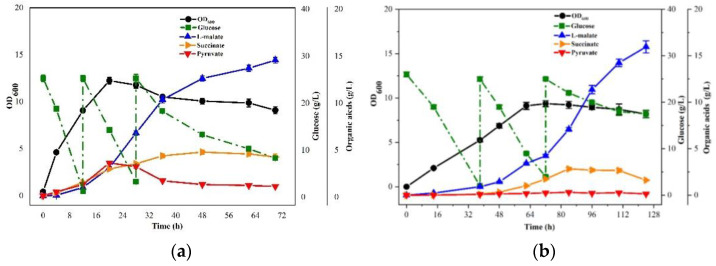
Time-course of glucose consumption, cell growth, and the organic acid production of *E. coli* E23 (**a**) and *E. coli* E23 (Δ*nuoA*) (**b**).

To investigate the effects of *nuoA* knockout on L-malate production, we compared the aerobic organic acid accumulation in E23 and E23 (Δ*nuoA*) by fed-batch cultivation in 5 L fermenters. As shown in Figure 5a,b, 14.4 g/L L-malate was accumulated from 63.0 g/L glucose with 4.12 g/L succinate and 1.0 g/L pyruvate in E23, and 16.1 g/L L-malate was accumulated from 52.5 g/L glucose with a trace of succinate and pyruvate accumulation in E23 (Δ*nuoA*). In this process, L-malate yield increased from 0.23 g/g to 0.31 g/g. In addition, E23 and E23 (Δ*nuoA*) took 22 h and 72 h to reach maximum cell density, and the highest OD_600_ also decreased from 11.3 to 9.4. These results indicated that cell growth was inhibited after knockout of *nuoA*. However, the malate productivity for E23 from 20 h to 70 h was 0.228 g/L·h, almost the same for E23 (Δ*nuoA*) from 72 h to 124 h at 0.226 g/L·h. That means that E23 (Δ*nuoA*) consumed less glucose with the same malate accumulation during the late period compared to E23. 

### 3.4. Limiting ATP Supply of E. coli E23 by the Introduction of the NOG System

The NOG system has the properties of zero ATP synthesis and zero CO_2_ emissions during aerobic cultivation from glucose to acetyl-CoA. To make the cells produce lower ATP, the NOG system was introduced by over-expressing the native *fbp* and *fxpk* from *Bifidobacterium adolescentis* to E23 (Δ*nuoA*), resulting in the generation of recombinant E23 (Δ*nuoA*, NOG). SDS-PAGE protein electrophoresis in Supplementary Figure S1 showed that bifunctional phosphoketolase (around 95 kDa) and fructose-1,6-bisphosphatase (around 45 kDa) were expressed successfully. To certify whether the NOG worked, the intracellular enzymatic activities of FPK, Ac-CoA, and ATP level were analyzed. As shown in Table 2, NOG function was confirmed because the AcCoA level was increased by 17.2%, and the ATP level decreased by 18.7% compared with E23 (Δ*nuoA*).

### 3.5. Metabolic Characteristics of E. coli E23 (ΔnuoA, NOG) in 5 L Fed-Batch Cultivation

**Figure 6:**
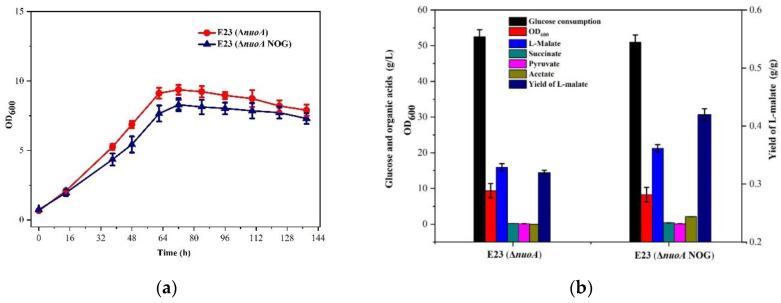
Effects of introduction of the NOG system to *E. coli* E23 (Δ*nuoA*) regarding glucose consumption and cell growth (**a**) and organic acids production (**b**).

To investigate the effects of NOG introduction on L-malate production, the aerobic organic acid accumulations of E23 (Δ*nuoA*) and E23 (Δ*nuoA*, NOG) were compared by fed-batch fermentation. As shown in Figure 6a,b, L-malate production by E23 (Δ*nuoA*, NOG) achieved 21.3 g/L with the yield of 0.43 g/g, which increased by 38.7% compared to 0.31 g/g yield of E23 (Δ*nuoA*) at the end of 138 h. Moreover, a slight reduction in cell growth was observed, and the maximum OD_600_ of E23 (Δ*nuoA*, NOG) was found to be lower than that of E23 (Δ*nuoA*). This phenomenon might be due to the introduction of a plasmid with two overexpressed genes. Consequently, it showed that a decrease in ATP levels could potentially enhance L-malate production by introducing the NOG system into the strain.

**Figure 7:**
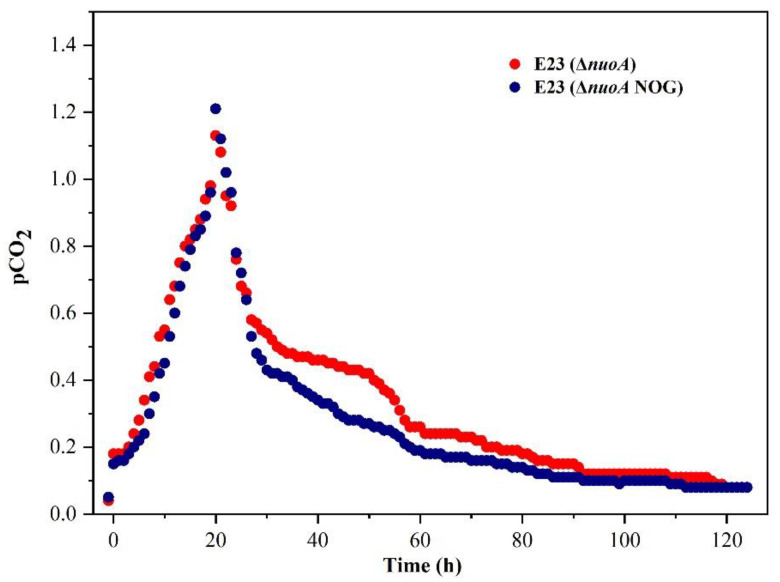
Effects of the introduction of the NOG system to *E. coli* E23 (Δ*nuoA*) regarding CO_2_ emissions rate.

To demonstrate the hypothesis that the NOG system could decrease CO2 emissions, the off-gas from the fermenter was measured by online CO_2_ detection. As shown in Figure 7, CO_2_ emissions could be divided into two phases. During the first phase, the CO_2_ emission rate of E23 (Δ*nuoA*) was comparable to that of E23 (Δ*nuoA*, NOG), while, in the second phase, the CO_2_ emission rate of E23 (Δ*nuoA*, NOG) was observed to be lower than that of E23 (Δ*nuoA*). This finding provides evidence that the introduction of the NOG system can effectively reduce CO_2_ emissions.

## 4. Discussions

Nutrient limitation regarding metabolic responses has been investigated in several studies by using information on isotope distribution data, resulting in improved accumulation of organic acids under limited conditions [27,28,29]. Additionally, it was also found that L-malate production with *Aspergillus oryzae* could be enhanced by a nitrogen regulation strategy [30]. Previous work also confirmed that limited nitrogen conditions decrease the ability of NAD^+^ regeneration, leading to the repression of the respiratory chain, thus triggering the overflow metabolism of L-malate with succinate as a carbon source [18]. 

In this study, we aim to produce L-malate aerobically using glucose as the carbon source with respect to *E. coli* E23, whose native promoters *aceBAK* and *ppc* were replaced with the *trc* promoter. Moreover, E23 was inactivated the NAD^+^-dependent and NADP^+^-dependent malic enzymes (*maeAB*), and the activity of isocitrate dehydrogenase was significantly decreased after long-term adaptive evolution. Thus, it is one of the candidates for L-malate production, with glucose as its carbon source, despite the relatively low L-malate yield of approximately 0.2 g/g. When E23 was cultivated under different concentrations of YE, we found that relatively lower YE facilitated L-malate accumulation, consistent with the findings when succinate was employed as the carbon source [18]. Conversely, when YE was added at more than 2 g/L, cells tended to proliferate, rather than overflow the metabolism, leading to a reduced accumulation of L-malate and succinate. Thus, nitrogen limitation was a key factor for the metabolites’ overflow metabolism. Moreover, transcriptional analysis revealed that *nuoA* and *atpA* were the most significantly down-regulated genes, which would decrease the NADH oxidation and the ATP synthesis in ETC. A reduction in NAD^+^ supplementation would inhibit the conversion of L-malate to OAA, as shown in Figure 1. Additionally, several studies have shown that metabolite production can benefit from decreasing ATP supplement, such as pyruvate [31] and glutamate [32], because lower ATP supplement could increase metabolic flux through central metabolic pathways by increasing ATP demand. In addition, reduced ATP production can lead to decreased cell growth, thereby increasing product yield during sugar fermentation [33]. Thus, we hypothesize that restricted NAD^+^ and ATP availability would enhance L-malate production under aerobic conditions.

To certify our speculation, *nuoA* in E23 was firstly deleted. The results showed that NAD^+^ regeneration was inhibited, and L-malate production was enhanced. Then, we tried to decrease the ATP generation by introducing the NOG system, which is an ATP-consuming pathway [34]. As shown in Figure 1, the overall reactions to synthesize pyruvate and acetyl-CoA using an EMP-based metabolism are as below:

Glucose → 2 Pyruvate + 2 NADH +2 ATP

Glucose → 2 acetyl-CoA + 2 CO_2_ + 4 NADH +2 ATP

These reactions provide crucial biosynthetic precursors, reducing equivalents and ATP for growth and maintenance. When NOG is introduced, it requires one ATP to phosphorylate glucose, and it forms three acetyl-CoAs per glucose:

Glucose + ATP → 3 acetyl CoA

Therefore, NOG is more efficient in the atom economy, and it does not generate CO_2_, but it cannot provide essential C3 metabolites to support the TCA cycle in glucose minimal medium. Thus, EMP and NOG were combined to flux the carbon source to TCA so as to balance cell growth and metabolism. Aiming for the accumulation of excessive L-malate, the glyoxylate pathway was activated, and the pathways of L-malate to pyruvate were inactivated. Moreover, instead of deleting the gene *mdh,* encoding malate dehydrogenase, the flux from L-malate to oxaloacetate was down-regulated by decreasing NAD^+^ supply via knockout of *nuoA*, which encodes the key component of ETC. The overall reactions for L-malate synthesis are shown below:

Glucose + ATP → 3 acetyl CoA

Glucose + 2 CO_2_ → 2 Oxaloacetate + 1 ATP

Oxaloacetate + 2 acetyl CoA + FAD^+^ → 2 L-malate + FADH

The net reaction for L-malate synthesis using this scheme below and ATP can be provided by further oxidation of FADH using the ETC. 

7 Glucose + 6 CO_2_ + 6 FAD^+^ + ATP → 12 L-malate + 6 FADH

Thus, the maximum theoretical mass yield is 1.27 g/g. However, the yield shown in Figure 6 only achieved 0.43 g/g, which was far from the theoretical maximum yield. The main factor was still the large amount of CO_2_ emissions, rather than the accumulation of coproducts, such as succinate, pyruvate, and acetate. To fully realize the potential of aerobic L-malate production by *E. coli*, CO_2_ emissions must be further decreased to enhance the L-malate yield by combining several strategies on account of the fact that, sometimes, no gradual increase in product accumulation was found upon step-wise introduction of metabolic modifications, but all genetic modifications had to be present simultaneously (Trichez et al. 2018). Here, the following recommendations are proposed to modify the pathways of L-malate production: (1) down-regulation of ICDH, based on fine tuning: isocitrate dehydrogenase is the main enzyme involving the production of CO_2_. Down-regulation of ICDH to a relative low activity would efficiently decrease the emissions of CO_2_; (2) further reduction of NAD^+^ and the ATP supplement was based on other components in the ETC: lower NAD^+^ decreases the flux, not only from L-malate to OAA, but also from pyruvate to Acetyl-CoA and CO_2_. Moreover, lower NAD^+^ could inhibit the activity of isocitrate dehydrogenase. Cell growth relies on ATP, and lower ATP could maintain cell density at a low level, and thus the total emissions of CO_2_ would decrease. 

## 5. Conclusions

The findings in this study suggest that limited NAD^+^ supply and reduced ATP level may prompt *E. coli* E23 to undergo incomplete oxidization of glucose, resulting in L-malate as the main product, rather than the respiratory pathway, to generate energy. The knockout of *nuoA* could decrease the efficiency of ETC and thus limit NAD^+^ supply. Moreover, the introduction of the NOG system can simultaneously reduce ATP production and CO_2_ emissions. Finally, E23 (Δ*nuoA* NOG) produced 21.3 g/L L-malate from glucose aerobically, with a yield of 0.43 g/g.

## Figures and Tables

**Table 1:** Strains that were used and constructed in this study.

Strains	Feature	Source or Reference
*E. coli* DH5α	Cloning host	Lab collection
*E. coli* E2	Evolved BL21(DE3), Δ*ppc*, *aceBAK:trc*	Li et al. 2013 [19]
*E. coli* E23	E2, *ppc*:trc, Δ*maeAB*	Chen’s Lab collection
*E. coli* E23 (Δ*nuoA*)	E23, Δ*nuoA*	This study
*E. coli* E23 (Δ*nuoA* NOG)	E23, Δ*nuoA*, harboring pTrc-*fxpk*-*fbp*	This study

**Table 2:** Analysis of intracellular acetyl-coA and ATP of the recombinants.

Strains	Acetyl-coA (nmol/g DCW)	ATP (nmol/g DCW)
*E. coli* E23 (Δ*nuoA*)	12.20 ± 0.76	1685 ± 416
*E. coli* E23 (Δ*nuoA* NOG)	14.30 ± 0.94	1370 ± 320

## Data Availability

Not applicable.

## Supplementary Materials

The following supporting information can be downloaded at: https://www.mdpi.com/article/10.3390/bioengineering10080881/s1, Table S1—Primers for *nuoA* knockout and identification. Table S2—Primers for construction of plasmid pTrc-*fxpk-fbp*. Figure S1—SDS-PAGE protein electrophoresis of recombinants of *E. coli* E23.
