# Supplementary material for: Cofactor Metabolic Engineering of Escherichia coli for Aerobic L-Malate Production with Lower CO2 Emissions
Source: Bioengineering (Basel). 2023 Jul 25;10(8):881. doi: 10.3390/bioengineering10080881 (PMC10451681; doi:10.3390/bioengineering10080881)
Supplement: Supplementary file 1 [file bioengineering-10-00881-s001.zip › bioengineering-2432535-supplementary.pdf]

Table S1 Primers for *nuoA* knockout and identification

Primers	DNA Sequence (5’-3 ’)
Primers for knock out	
Target-S	GATGACTAGTATTATACCTAGGACTGAG
Target *nuo*-A	CTAGACTAGTCTGAAGTCATCGCTCATCACGTTTTAGAGCTAGAAATAG CAAG
nuo-5-S	GTCGGTGCTTTTTTTGAATTCATAAAGCAGATATTCCATGGCGAC
nuo-5-A	GATCTATGCGGGTGAGCGTATAATGTAGCAGGTGATTTTTTTCAGGC
nuo-3-S	GCCTGAAAAAAATCACCTGCTACATTATACGCTCACCCGCATAGATC
nuo-3-A	CAGGTCGACTCTAGAGAATTCTAGATTCCTGCAACAGCATCAGTG
nuo-V-S	CCATCGTCCTTCAGCGTTTA
nuo-V-A	AGGTTAGTTACGGCAATGCGTTC

Table S2 Primers for construction of plasmid pTrc-*fxpk*-*fbp*

Primers	DNA Sequence (5’-3 ’)
fxpk-S	TTTCACACAGGAAACAGACCATGGAATTCATGACGAGTCCTGTTATT GGCACC
fxpk-A	GCATGCCTGCAGGTCGACTCTAGACTCGTTATCGCCAGCGGTT
fbp-S	CCGCCGAACCGCTGGCGATAACGAGATGAAAACGTTAGGTGAATTT ATTGTCG
fbp-A	GCATGCCTGCAGGTCGACTCTAGATTACGCGTCCGGGAACTCA

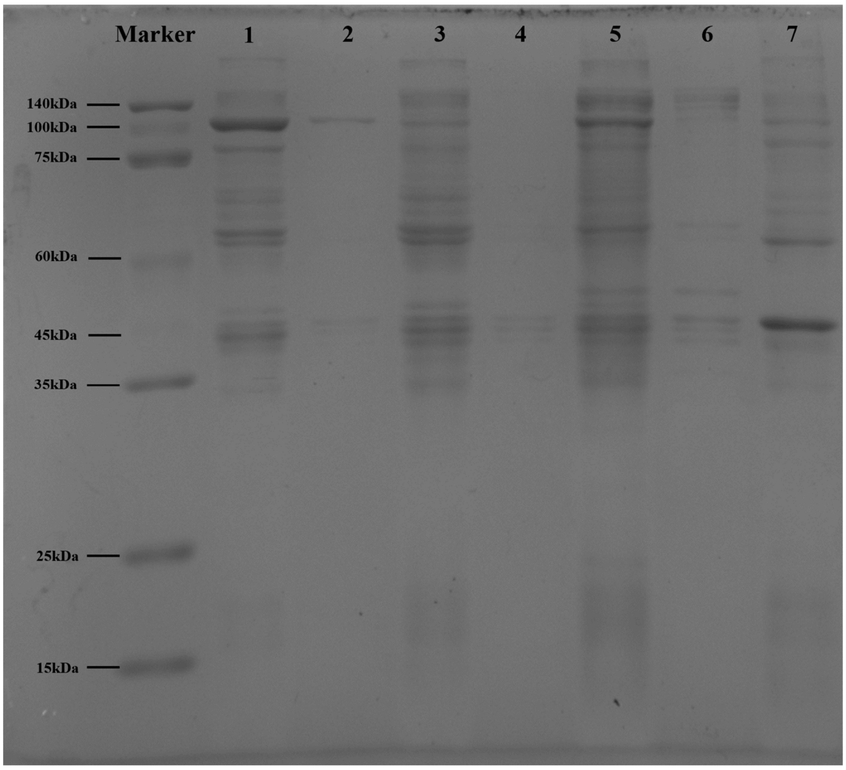

Figure S1 SDS-PAGE protein electrophoresis of recombinants of *E. coli* E23. Sample 1 and 2 are the supernatant and pellets of E23 (pTrc-*fxpk*). Sample 3 and 4 are the supernatant and pellets of E23 (pTrc99a). Sample 5 and 6 are the supernatant and pellets of E23 (pTrc-*fxpk*-*fbp*). Sample 7 is the supernatant of E23 (pTrc-*fbp*).
